# Psychological burden and quality of life in newly diagnosed inflammatory bowel disease patients

**DOI:** 10.3389/fpsyg.2024.1334308

**Published:** 2024-01-29

**Authors:** Purificación Bernabeu, Olivia Belén-Galipienso, Carlos van-der Hofstadt, Ana Gutiérrez, Lucía Madero-Velázquez, Gloria García del Castillo, Mariana-Fe García-Sepulcre, Mariam Aguas, Pedro Zapater, Jesús Rodríguez-Marín, María-Teresa Ruiz-Cantero, José I. Cameo, Rodrigo Jover, Laura Sempere

**Affiliations:** ^1^Health Psychology Department, Dr. Balmis General University Hospital, Alicante Institute for Health and Biomedical Research (ISABIAL), Alicante, Spain; ^2^Gastroenterology Department, Dr. Balmis General University Hospital, Alicante Institute for Health and Biomedical Research (ISABIAL), Alicante, Spain; ^3^Department of Health Psychology, Miguel Hernández University, Alicante, Spain; ^4^Centro de Investigación Biomédica en Red de Enfermedades Hepáticas y Digestivas (CIBEREHD), Instituto de Salud Carlos III, Madrid, Spain; ^5^Gastroenterology Department, University Hospital, San Juan, Alicante, Spain; ^6^Gastroenterology Department, General University Hospital, Elche, Alicante, Spain; ^7^Gastroenterology Department, Hospital Universitario y Politécnico la Fe, Valencia, Spain; ^8^Clinical Pharmacology Department, Dr. Balmis General University Hospital, Alicante Institute for Health and Biomedical Research (ISABIAL), Alicante, Spain; ^9^Department of Clinical Medicine, University Miguel Hernández de Elche, Alicante, Spain; ^10^Public Health Department, University of Alicante, Alicante, Spain; ^11^Centro de Investigación Biomédica en Red de Epidemiología y Salud Pública (CIBERESP), Madrid, Spain

**Keywords:** anxiety, depression, quality of life, newly diagnosed IBD, Crohn’s disease, ulcerative colitis

## Abstract

**Objective:**

Psychological factors, such as stress, anxiety, and depression, are frequently related to inflammatory bowel disease (IBD). However, few studies have examined these factors in patients newly diagnosed with IBD. The aim of the present study was to test the psychological burden in patients with a recent diagnosis of IBD and the factors related to this psychological burden.

**Methods:**

We performed a prospective, multi-center, observational study in patients with a new diagnosis of IBD (≤6 months). The patients were recruited from four different Spanish hospitals. Clinical and demographic characteristics were collected. Patients were evaluated using the Hospital Anxiety and Depression Scale and quality of life questionnaire for patients with inflammatory bowel disease (IBDQ-32). The Scale of Stress Perceived by the Disease was used to assess stressful life events.

**Results:**

We included 156 patients newly diagnosed with IBD [69 women; 80 Crohn’s disease (CD) and 76 ulcerative colitis (UC)], with a mean age of 42.3 (SD 16.21) years. A total of 37.2% of patients had symptoms of anxiety and 17.3% had symptoms of depression. Quality of life was affected in 30.1% of patients. Factors related to anxiety in early IBD were being a woman and having CD. The only factor related to depression was the presence of comorbidity. Being a woman and having suffered previous stressful life events were factors related to impaired quality of life.

**Conclusion:**

Anxiety, depression, and impaired quality of life are frequent in patients with a recent diagnosis of IBD. This psychological burden is greater in women.

## Introduction

Inflammatory bowel disease (IBD), including ulcerative colitis (UC) and Crohn’s disease (CD), is a chronic condition with considerable prevalence worldwide. Although the causes of the disease are unknown, there is a general consensus that it is the result of multiple interactions between genetic and environmental factors (Rogler et al., 2018). Among the environmental factors, available evidence suggests that stress, anxiety, and depression may contribute to the development and progression of IBD (Oligschlaeger et al., 2019; Sun et al., 2019). However, few studies have clearly identified it as an underlying factor in the multicausal etiology, and results have been contradictory (Li et al., 2004; Lerebours et al., 2007; Maunder and Levenstein, 2008; Włodarczyk et al., 2016; Krishna et al., 2018). Moreover, there are no studies describing the presence of emotional factors early after IBD diagnosis. Diagnosis of a chronic condition can have an important emotional impact affecting quality of life (Flanagan et al., 2017). The presence of anxiety and depression after diagnosis could play a role in the future development of the disease since it has been described that flares of IBD may be stress-induced (Araki et al., 2020). On the other hand, the literature on IBD suggests that the disease itself makes patients more susceptible to other psychological problems, such as anxiety, depression, or post-traumatic stress disorder (Choi et al., 2019; Taft et al., 2019; Irving et al., 2021; Ludvigsson et al., 2021). A meta-analysis by Barberio et al. (2021) showed an overall prevalence of anxiety and depression symptoms of 32.1 and 25.2%, respectively, in patients with IBD.

The main objective of this study was to assess the psychological burden suffered by newly diagnosed IBD patients and to examine factors potentially related to emotional problems in these patients.

## Materials and methods

### Study population

We conducted an observational multicenter study in a cohort of patients with IBD diagnosed from January 2018 to March 2020 in four hospitals in the Valencian Community of Spain. Consecutive patients diagnosed with IBD in the last 6 months who were older than 18 years of age and had clinical symptoms at onset were included. The inclusion criterion was a diagnosis of symptomatic CD or UC according to European Crohn’s and Colitis Organization (ECCO) criteria. The exclusion criteria were intellectual disability, dementia, major psychiatric disorders, and the presence of a language barrier.

### Sample size calculation

The sample size was calculated to perform a multivariate logistic regression analysis in which five independent variables were included and 40% of the patients showed the event analyzed (Peduzzi et al., 1996) with a significance level of 0.05 and a power of 0.8. Based on these results, the required sample size was estimated to be 125 patients.

### Data collection and variables

Clinical and demographic characteristics, disease activity, biomarkers, and hemoglobin were assessed at the time of IBD diagnosis. Clinical variables were collected from electronic medical records. Disease phenotype was classified according to the Montreal Classification (Silverberg et al., 2005). The cutoff value differentiating mild from moderate–severe IBD was set to 8 for Harvey-Bradshaw index (HBI) (<8 indicates mild CD) and 6 for Mayo Score (MS) (<6 indicates mild UC). C-reactive protein (CRP) and fecal calprotectin (FC) were used as biomarkers. The cut-off for CRP was 0.8 mg/L and for FC 250 mcg/g. According to European Crohn’s and Colitis Organization (ECCO) guidelines and the World Health Organization (WHO), anemia is defined as hemoglobin <12.0 g/dL in female and < 13.0 g/dL in male (Dignass et al., 2015). Comorbidity was characterized as coexisting diseases or conditions that affect an individual’s physiological reserve condition or require chronic treatment.

Newly diagnosed patients from the four participant hospitals were invited to respond to a semi-structured interview conducted by a psychologist (PB). Sociodemographic information regarding sex, age, education level [split into low level (secondary school or lower) and high level (college or higher)] employment status, and marital status were obtained during the interview.

Psychological variables were measured using appropriate tests and scales. Stressful life events were assessed using the Social Readjustment Rating Scale (SRRS; Holmes and Rahe, 1967), which contains a list of life events that are considered potentially stressful. In this test, scores ≥150 are considered to indicate a stress-induced health risk.

The Hospital Anxiety and Depression Scale (HADS; Zigmond and Snaith, 1983) was used to assess the existence of symptoms of anxiety (HADS-A) and depression (HADS-D). A score > 7 on each subscale indicates high level of anxiety or depression.

Quality of life was measured using the Spanish version of the specific Inflammatory Bowel Disease Questionnaire (IBDQ-32; Masachs et al., 2007), which contains four dimensions (intestinal symptoms, emotional function, social function, and systemic symptoms). A score < 160 reflects a suboptimal quality of life.

### Primary outcome

The main objective of our study was to assess the psychological burden suffered by patients recently diagnosed with IBD and to determine which of the analyzed sociodemographic, disease-related, or treatment-related factors are associated with anxiety, depression, or impaired quality of life.

### Statistical analysis

Descriptive statistics were used to examine the baseline characteristics. Variables with a probable relationship to the presence of symptoms of anxiety and depression and quality of life were evaluated in a univariate analysis. Variables with a value of *p* < 0.05 in the univariate analysis were included in a multivariable logistic regression model. The significance level for all variables was set at *p* < 0.05. We used the SPSS statistical package for Windows, version 25.0.

## Results

A total of 183 patients were invited to participate in this study. Twenty-seven patients were excluded: eight did not meet the inclusion criteria and 19 declined to participate. Finally, a total of 156 patients were included in the study: 69 (44.2%) women and 87 (55.8%) men. The mean age was 42.30 (SD 16.21) years. Seventy-six (48.7%) patients had a diagnosis of UC and 80 (51.3%) a diagnosis of CD. All 156 patients completed the psychological evaluation. Additional demographic and clinical characteristics at inclusion are provided in Table 1.

**Table 1:** Clinical and demographic characteristics of the study population.

*N* = 156	*n* (%)
Sex
Men	87 (55.8)
Women	69 (44.2)
Median age, years (IQR)	42 (28–55)
Disease type
UC	76 (48.7)
CD	80 (51.3)
Median BMI, kg/m^2^ (IQR)	23.9 (21.6–27.8)
Current smoker
Smoker	27 (17.3)
Former smoker	61 (39.1)
Non-smoker	68 (43.6)
Marital status
Married/partner	94 (60.3)
Divorced/Single/ Widowed	62 (39.7)
Education
Low level (secondary school or lower)	107 (68.6)
High level (college or higher)	49 (31.4)
Have children	92 (59)
Active employee	83 (53.2)
Comorbidity	66 (42.3)
Previous history of MAD	14 (9)
CD Montreal classification (*n*: 80)
Age group	
A2: 17–40 years	44 (55)
A3: ≥40 years	36 (45)
Location of Crohn’s (>1 location possible)
L1: Ileal	42 (52.5)
L2: Colonic	12 (15)
L3: Ileocolonic	25 (31.3)
L4: Upper GI	6 (7.5)
Crohn’s behavior
B1: Inflammatory	63 (78.8)
B2: Stricturing	11 (13.8)
B3: Penetrating	6 (7.5)
Perianal involvement	5 (6.3)
UC Montreal classification (*n*: 76)
E1: Proctitis	22 (28.9)
E2: Left-sided colitis	30 (39.5)
E3: Extensive colitis	24 (31.6)
Active IBD at diagnosis
Mild	93 (59.6)
Moderate/Severe	63 (40.4)
Median CRP, mg/L (IQR)	0.7 (0.2–4.5)
Median fecal calprotectin, μg/g (IQR)	619 (92–1,718)
Anemia	47 (30.1)
Use of mesalazine	110 (70.5)
Use of steroids	104 (66.7)
Use of thiopurinics	34 (21.8)
Use of biologics	37 (23.7)
IBD-related surgery	6 (3.8)
Hospitalization	71 (45.5)
EIMs	19 (12.2)

Values are *n* (%) unless otherwise noted. IQR, Interquartile range; UC, Ulcerative colitis; CD, Crohn’s disease; BMI, Body mass index; MAD, Mood and/or anxiety disorders; GI, gastrointestinal; IBD, Inflammatory bowel disease; CRP, C-reactive protein; and EIMs, Extra-intestinal manifestations.

**Figure 1:**
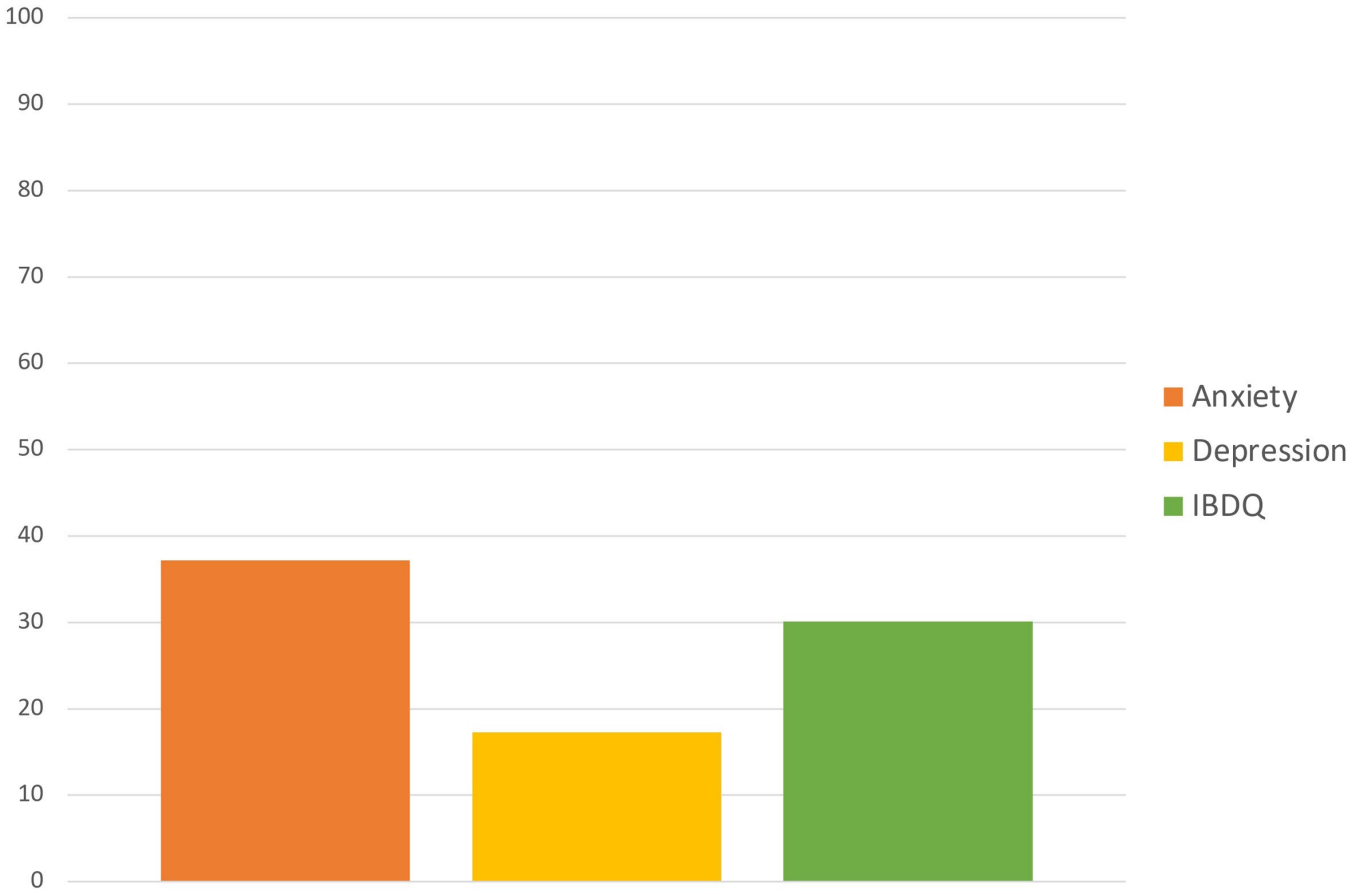
Proportion of patients with anxiety, depression, or quality of life impairment.

A total of 120 patients (76.9%) had high scores in stressful life events. Regarding the psychometric evaluation, a total of 58 (37.2%) patients had high anxiety symptoms at the onset of the disease. A total of 27 (17.3%) patients had high depression symptoms at the onset of the disease. Forty-seven (30.1%) patients had impaired quality of life on the IBDQ. The proportion of patients with impairment in these psychological variables is shown in Figure 1.

### Factors related to anxiety, depression, and impaired quality of life

Several factors were associated with anxiety in the univariate analysis (Supplementary Table 1). In the multivariate analysis, being a woman (odds ratio [OR] 4.85; 95% confidence interval [CI] 2.21–10.6), having CD (OR 3.5; 95% CI 1.54–8.2), and the use of biologics (OR 0.139; 95%CI 0.04–0.40), in this case, as a protective factor, maintained an independent relationship with anxiety (Table 2).

**Table 2:** Factors associated with anxiety (*n*: 58) in early IBD.

		*Univariate analysis*			*Multivariate analysis*		
	HADS-Anxiety >7/total *n* anxiety/*n* total (%)	OR	95% confidence interval	*p*	Adjusted OR	95% confidence interval	*p*
Sex							
Women	38/69 (55.1)	4.1	2.1–8.2	**<0.001**	4.85	2.21–10.6	**<0.001**
Men	20/87 (23.0)	1					
Disease type							
CD	39/80 (48.8)	2.85	1.45–5.63	**0.002**	3.5	1.54–8.2	**0.003**
UC	19/76 (25.0)	1					
Current smoker							
Yes	15/27 (55.6)	2.5	1.08–5.81	**0.030**	1.99	0.71–5.61	0.190
No	43/129 (33.3)	1					
Use of biologics							
Yes	7/37 (18.9)	0.31	0.13–0.76	**0.009**	0.139	0.04–0.40	**<0.001**
No	51/119 (42.9)	1					
SRRS ≥ 150							
Yes	51/120 (42.5)	3.06	1.24–7.54	**0.012**	1.9	0.698–5.19	0.208
No	7/36 (19.4)	1					

Only variables significantly related with anxiety in the univariate analysis are included in this table. CD, Crohn’s disease; UC, Ulcerative colitis; IBD, Inflammatory bowel disease; HADS, Hospital anxiety and depression scale; and SRRS, Social Readjustment Rating Scale. Bold values indicate statistical significance at the *p* < 0.05 level.

Regarding depression, several factors in the univariate analysis were associated with early IBD in our cohort of patients (Supplementary Table 2). However, in the multivariate analysis, only comorbidity remained independently associated with depression (OR 3.32; 95% CI 1.06–10.3; Table 3).

**Table 3:** Factors associated with depression (*n*: 27) in early IBD.

		*Univariate analysis*			*Multivariate analysis*		
	HADS Depression >7/total *n* depression/*n* total (%)	OR	95% confidence interval	*p*	Adjusted OR	95% confidence interval	*p*
Age
<40 years	8/73 (11)	1	0.98–5.9	**0.049**	0.91	0.26–3.11	0.881
≥40 years	19/83 (22.9)	2.41					
Disease type
CD	20/80 (25.0)	3.29	1.30–8.30	**0.009**	2.64	0.84–8.25	0.095
UC	7/76 (9.2)	1					
Have children
Yes	21/92 (22.8)	2.86	1.08–7.55	**0.029**	1.98	0.55–7.11	0.295
No	6/64 (9.4)	1					
Education
Low level	24/107 (22.4)	4.43	1.27–15.52	**0.012**	3.06	0.73–12.8	0.126
High level	3/49 (6.1)	1					
Active employment
Yes	8/83 (9.6)	1	1.34–8.09	**0.007**	1.87	0.64–5.1	0.255
No	19/73 (26)	3.3					
Comorbidity
Yes	19/66 (28.8)	4.14	1.68–10.2	**0.001**	3.32	1.06–10.3	**0.038**
No	8/90 (8.9)	1					
History of MAD
Yes	6/14 (42.9)	4.32	1.36–13.7	**0.008**	1.6	0.4–6.29	0.498
No	21/142 (14.8)	1					
Active IBD
Mild	17/63 (27)	3.06	1.29–7.25	**0.009**	2.35	0.81–6.86	0.116
Moderate to severe	10/93 (10.8)	1					

Only variables significantly related with depression in the univariate analysis are included in this table. HADS, Hospital anxiety and depression scale; CD, Crohn’s disease; UC, Ulcerative colitis; MAD, Mood and/or anxiety disorders; and IBD, Inflammatory bowel disease. Bold values indicate statistical significance at the *p* < 0.05 level.

Several factors were associated with quality of life in the univariate analysis (Supplementary Table 3). However, in the multivariate analysis, only being a woman (OR 3.04; 95% CI 1.39–6.53) and having experienced stressful events on the SRRS, with scores ≥150 (OR 3.55; 95% CI 1.08–11.6), were independently related to significant impairment in perceived quality of life (Table 4).

**Table 4:** Factors associated with poor quality of life (*n*: 47) in early IBD.

		*Univariate analysis*			*Multivariate analysis*		
	IBDQ32 < 160/total *n* low IBDQ/*n* total (%)	OR	95% confidence interval	*p*	Adjusted OR	95% confidence interval	*p*
Sex							
Women	30/69 (43.5)	3.17	1.55–6.46	**0.001**	3.04	1.39–6.53	**0.005**
Men	17/87 (19.5)	1					
Disease type							
CD	34/80 (42.5)	3.58	1.7–7.5	**0.001**	1.64	0.6–4.49	0.332
UC	13/76 (17.1)	1					
BMI							
<25	33/88 (37.5)	1	0.2–0.89	**0.022**	0.7	0.31–1.59	0.402
≥25	14/68 (20.6)	0.43					
Use of mesalazine							
Yes	25/110 (22.7)	0.321	0.15–0.67	**0.002**	0.51	0.19–1.4	0.194
No	22/46 (47.8)	0.321					
Use of steroids							
Yes	38/104 (36.5)	2.75	1.2–6.25	**0.014**	1.76	0.69–4.46	0.230
No	9/52 (17.3)	1					
SRRS ≥ 150							
Yes	43/120 (35.8)	4.47	1.48–13.48	**0.005**	3.55	1.08–11.6	**0.036**
No	4/36 (11.1)	1					

Only variables significantly related with impairment in quality of life in the univariate analysis are included in this table. CD, Crohn’s disease; UC, Ulcerative colitis; BMI, Body mass index; IBDQ, Quality of life questionary for IBD; and SRRS, Social readjustment rating scale. Bold values indicate statistical significance at the *p* < 0.05 level.

## Discussion

The main finding in our study is that patients newly diagnosed with IBD suffer a significant psychological burden, which is reflected in anxiety and depression, and have a high impairment of quality of life as measured by the IBDQ. This psychological burden is influenced by different factors, for instance, female gender is associated with presence of anxiety and impaired quality of life. Our results show the special frailty that IBD patients suffer at the moment of the diagnosis, especially women, and underscores the importance of establishing appropriate and specific early intervention guidelines to alleviate the psychological burden in these newly diagnosed patients.

The early months after diagnosis are particularly challenging for patients with chronic conditions, such as IBD, because of the complexity of the medical information, testing, medication adjustments, and therapeutic strategies being discussed and implemented (Engel et al., 2021). Thus, psychological support is an important dimension of patient care, particularly early in the disease course. Resources received by patients with IBD from a medical professional during an educational or psychological support session have the potential to positively impact how a patient copes with the diagnosis (Engel et al., 2021). Our results highlight the role of early psychological assessments and interventions in preventing the exacerbation of disease activity in IBD and improving the patient’s quality of life (Farbod et al., 2022).

Both depression and anxiety are significant experiences in IBD. Whether these are a consequence of the disease or an active contributor to the disease remains controversial (Eugenicos and Ferreira, 2021). Previous studies (Kurina et al., 2001) have shown that the depression or anxiety is a reaction to the IBD, with a high prevalence in the year after diagnosis. These disturbances can be caused by the physical symptoms of the IBD itself or, perhaps, by the side effects from, for example, corticosteroids, a common treatment for IBD. Our data show that emotional impairment is frequently present as early as 3–6 months after diagnosis; therefore, early intervention is important in this context. IBD presents a significant impact on mental health. Feelings of shame, isolation, and body dissatisfaction, which compromise psychosocial functioning, are often reported as being increased by the experience of having the disease (Eugenicos and Ferreira, 2021). In addition, in young people, a recent diagnosis of IBD represents a dramatic change in self-perception and acceptance, as well as a change in life perspectives.

In this study, we also identified risk factors for early emotional impairment. These risk factors are being a woman, having CD, presence of comorbidities, and having experienced stressful events previous to diagnosis. Moreover, we found that these susceptibility factors are different for anxiety, depression, and quality of life. Based on our results, although psychological support should be offered to all individuals with a recent diagnosis of IBD, people with special susceptibility to emotional disorders should be intensively monitored for emotional impairment and prioritized for early psychological support. Gender differences in the course and presentation of IBD have already been reported (Greuter et al., 2020; Sempere et al., 2023). Women are more prone to develop anxiety and mood disorders than men (Asher et al., 2017) However, this specific frailty found in women with IBD regarding psychological impairment should be specifically addressed. Moreover, it is important to investigate the potential causes of this different behavior during the early course of the disease between men and women. Symptoms of anxiety and depression were more common in patients with CD than in those with UC. Similarly, some studies have observed an association between symptoms of common mental disorders and CD, but not UC (Nordin et al., 2002; Barberio et al., 2021). These data indicate that these mental disorders may be present at early diagnosis. It has also been hypothesized that this could be the result of more severe somatic symptoms in CD (Nordin et al., 2002).

Usual IBD care appropriately targets both symptoms and inflammation. However, it is important to consider that the burden of symptoms is determined by how an individual thinks and feels about their symptoms (Eldridge et al., 2020). Gracie et al. (2018) demonstrated a bi-directional relationship between disease activity and psychological disorders in CD and UC, highlighting the potential impact of brain-gut axis activity on the natural history of IBD. Different studies have demonstrated the role of psychological therapies on quality of life in patients with IBD (Knowles et al., 2013; Neilson et al., 2016; Gracie et al., 2017, 2018; Bernabeu et al., 2021). These results reinforce the need for integration therapies that target inflammatory disease activity with novel interventions that aim to improve psychological well-being in patients with IBD. Currently, the access to psychological assistance by IBD patients remains difficult, despite evidence that engagement in treatment is higher if mental health support is integrated into IBD services. Integration of psychologists into the IBD team will result in the team being better equipped to work with the complexity of the inter-related biopsychosocial aspects of IBD (Eldridge et al., 2020).

The strengths of our study are the rigorous selection of patients with a recent diagnosis of IBD that allows them to be accurately evaluated in light of the relationship between emotional factors and time from diagnosis. Patients were involved in a multidisciplinary team including gastroenterologists, psychologists, and specialized IBD nurses. Sample size was calculated to include an adequate number of cases to demonstrate our hypothesis. Patients were closely monitored during this early period. Moreover, the multicenter characteristics of the study allowed us to avoid ascertainment bias related to particular models of care. The limitations of our study are a lack of a more extensive psychometric evaluation that allows us to study inter-related influences between different psychological factors, such as stress and anxiety-depression or health-related quality of life. Also, the high rate of steroid therapy, due to a recent onset of the disease might have an influence on the findings. In addition, although multicentric, all of the participating hospitals are in the same geographical area, making it difficult to generalize our findings.

In summary, our study shows that newly diagnosed IBD patients have elevated levels of anxiety and depression, and their quality of life is affected. We have also identified specific risk factors for these psychological disturbances, and being a woman is a frequently related factor. Early detection of anxious or depressive symptomatology in patients with IBD, as well as its treatment in the initial stages of the disease, would be an important factor for managing the evolution of the disease in a more adaptative way. Our study highlights the importance of psychological evaluation and intervention in IBD patients from the onset of the disease and remarks on the role of sex and gender in the emotional impact of this disease. Furthermore, knowing to which factors this symptomatology is related would help with more effective prevention.

## Data availability statement

The raw data supporting the conclusions of this article will be made available by the authors, without undue reservation.

## Ethics statement

The studies involving humans were approved by the Ethics Committee of HGUA-ISABIAL (PI 2018/047). The studies were conducted in accordance with the local legislation and institutional requirements. The participants provided their written informed consent to participate in this study.

## Author contributions

PB: Conceptualization, Data curation, Formal Analysis, Investigation, Methodology, Project administration, Resources, Software, Supervision, Validation, Visualization, Writing – original draft, Writing – review & editing. OB-G: Investigation, Resources, Validation, Visualization, Writing – review & editing, Writing – original draft, Formal Analysis. CH: Conceptualization, Data curation, Investigation, Methodology, Project administration, Resources, Validation, Writing – review & editing. AG: Writing – review & editing. LM-V: Writing – review & editing. GG: Writing – review & editing. M-FG-S: Writing – review & editing. MA: Writing – review & editing. PZ: Data curation, Methodology, Supervision, Writing – review & editing. JR-M: Writing – review & editing. M-TR-C: Writing – review & editing. JC: Data curation, Investigation, Methodology, Resources, Writing – review & editing. RJ: Supervision, Validation, Writing – review & editing. LS: Conceptualization, Data curation, Formal Analysis, Funding acquisition, Investigation, Methodology, Project administration, Resources, Software, Supervision, Validation, Writing – original draft, Writing – review & editing.
