# Supplementary material for: Psychological burden and quality of life in newly diagnosed inflammatory bowel disease patients
Source: Front Psychol. 2024 Jan 29;15:1334308. doi: 10.3389/fpsyg.2024.1334308 (PMC10859525; doi:10.3389/fpsyg.2024.1334308)
Supplement: Supplementary file 1 [file Table_1.docx]

**Table supplementary 1.** Factors associated with anxiety (n:58) in early IBD.

|  | **Anxiety** | | | |
| --- | --- | --- | --- | --- |
|  | **HADS Anxiety >7/ total**  n anxiety/ n total (%) | **OR** | **95% confidence interval** | ***P*** |
| Sex  Women  Men | 38 / 69 (55.1)  20 / 87 (23.0) | 4.1  1 | 2.1-8.2 | **< 0.001** |
| Age  <40 years  ≥40 years | 29 / 73 (39.7)  29 / 83 (34.9) | 1  0.81 | 0.42­–1.56 | 0.537 |
| Disease Type  CD  UC | 39 / 80 (48.8)  19 / 76 (25.0) | 2.85  1 | 1.45­–5.63 | **0.002** |
| BMI  <25  ≥25 | 36 / 88 (40.9)  22 / 68 (32.4) | 1  0.69 | 0.35­–1.34 | 0.273 |
| Current smoker  Yes  No | 15 / 27 (55.6)  43 / 129 (33.3) | 2.5  1 | 1.08­–5.81 | **0.030** |
| Marital status  Married /partner  Divorced/ Single/ Widowed | 39 / 94 (41.5)  19 / 62 (30.6) | 1.6  1 | 0.81-3.2 | 0.170 |
| Children  Yes  No | 37 / 92 (40.2)  21 / 64 (32.8) | 1.4  1 | 0.7-2.7 | 0.347 |
| Education  Low level  High level | 41 / 107 (38.3)  17 / 49 (34.7) | 1.2  1 | 0.58-2.4 | 0.664 |
| Active employment  Yes  No | 30 / 83 (36.1)  28 / 73 (38.4) | 1  1.10 | 0.57–2.10 | 0.775 |
| Comorbidity  Yes  No | 28 / 66 (42.4)  30 / 90 (33.3) | 1.47  1 | 0.77-2.84 | 0.246 |
| Previous history of MAD  Yes  No | 8 / 14 (57.1)  50 / 142 (35.2) | 2.45  1 | 0.81-7.47 | 0.105 |
| Active IBD  Mild  Moderate to Severe | 25 / 63 (39.7)  33 / 93 (35.5) | 1.20  1 | 0.62-2.31 | 0.594 |
| EIM  Yes  No | 10 / 19 (52.6)  48 / 137 (35.0) | 2.06  1 | 0.78-5.42 | 0.137 |
| Anemia  Yes  No | 20 / 47 (42.6)  38 / 109 (34.9) | 1.38  1 | 0.69-2.79 | 0.362 |
| CRP > 8 mg/L  Yes  No | 29 / 77 (37.7)  29 / 79 (36.7) | 1.04  1 | 0.54-1.99 | 0.902 |
| Fecal calprotectin > 250 µg/g  Yes  No | 34 / 98 (34.7)  24 / 58 (41.4) | 0.75  1 | 0.39-1.47 | 0.404 |
| Use of mesalazine  Yes  No | 39 / 110 (35.5)  19 / 46 (41.3) | 0.78  1 | 0.39-1.58 | 0.491 |
| Use of steroids  Yes  No | 44 / 104 (42.3)  14 / 52 (26.9) | 1.99  1 | 0.96-4.11 | 0.061 |
| Thiopurines  Yes  No | 12 / 34 (35.3)  46 /122 (37.7) | 0.90  1 | 0.40-1.99 | 0.797 |
| Use of biologics  Yes  No | 7 / 37 (18.9)  51 / 119 (42.9) | 0.31  1 | 0.13-0.76 | **0.009** |
| IBD-related surgical history  Yes  No | 2 / 6 (33.3)  56 / 150 (37.3) | 0.84  1 | 0.15-4.73 | 1 |
| Hospitalization  Yes  No | 29 / 71 (40.8)  29 / 85 (34.1) | 1.33  1 | 0.69-2.56 | 0.387 |
| SRRS ≥ 150  Yes  No | 51 / 120 (42.5)  7 / 36 (19.4) | 3.06  1 | 1.24-7.54 | **0.012** |

CD, Crohn’s disease; UC, ulcerative colitis, BMI, Body Mass Index; MAD, mood and/or anxiety disorders; IBD, inflammatory bowel disease; EIM, extraintestinal manifestations; CRP, C-reactive protein;SRRS Social Readjustment Rating Scale; HADS, hospital anxiety and depression scale
