# Supplementary material for: Psychological burden and quality of life in newly diagnosed inflammatory bowel disease patients
Source: Front Psychol. 2024 Jan 29;15:1334308. doi: 10.3389/fpsyg.2024.1334308 (PMC10859525; doi:10.3389/fpsyg.2024.1334308)
Supplement: Supplementary file 2 [file Table_2.docx]

|  | **HADS Depression >7 / total**  n depression / n total (%) | **OR** | **95% confidence interval** | ***P*** |
| --- | --- | --- | --- | --- |
| Sex  Women  Men | 15 / 69 (21.7)  12 / 87 (13.8) | 1.73  1 | 0.75-4.00 | 0.193 |
| Age  <40 years  ≥40 years | 8 / 73 (11)  19 / 83 (22.9) | 1  2.41 | 0.98-5.90 | **0.049** |
| Disease Type  CD  UC | 20 / 80 (25.0)  7 / 76 (9.2) | 3.29  1 | 1.30–8.30 | **0.009** |
| BMI  <25  ≥25 | 13 / 88 (14.8)  14 / 68 (20.6) | 1  1.50 | 0.65–3.44 | 0.341 |
| Current smoker  Yes  No | 5 / 27 (18.5)  22 / 129 (17.1) | 1.10  1 | 0.38-3.23 | 0.855 |
| Marital status  Married /partner  Divorced/ Single/ Widowed | 19 / 94 (20.2)  8 / 62 (12.9) | 1.71  1 | 0.70-4.19 | 0.238 |
| Children  Yes  No | 21 / 92 (22.8)  6 / 64 (9.4) | 2.86  1 | 1.08-7.55 | **0.029** |
| Education  Low level  High level | 24 / 107 (22.4)  3 / 49 (6.1) | 4.43  1 | 1.27-15.52 | **0.012** |
| Active employment  Yes  No | 8 / 83 (9.6)  19 / 73 (26) | 1  3.30 | 1.34-8.09 | **0.007** |
| Comorbidity  Yes  No | 19 / 66 (28.8)  8 / 90 (8.9) | 4.14  1 | 1.68-10.2 | **0.001** |
| Previous history of MAD  Yes  No | 6 / 14 (42.9)  21 / 142 (14.8) | 4.32  1 | 1.36-13.7 | **0.008** |
| Active IBD  Mild  Moderate to Severe | 17 / 63 (27)  10 / 93 (10.8) | 3.06  1 | 1.29-7.25 | **0.009** |
| EIM  Yes  No | 5 / 19 (26.3)  22 / 137 (16.1) | 1.87  1 | 0.61-5.71 | 0.268 |
| Anemia  Yes  No | 8 / 47 (17.0)  19 / 109 (17.4) | 0.97  1 | 0.39-2.41 | 0.950 |
| CRP > 8 mg/L  Yes  No | 11 / 77 (14.3)  16 / 79 (20.3) | 0.66  1 | 0.28-1.52 | 0.325 |
| Fecal calprotectin > 250 µg/g  Yes  No | 17 / 98 (17.3)  10 / 58 (17.2) | 1.01  1 | 0.43-2.38 | 0.987 |
| Use of mesalazine  Yes  No | 18 / 110 (16.4)  9 / 46 (19.6) | 0.80  1 | 0.33-1.95 | 0.630 |
| Use of steroids  Yes  No | 19 / 104 (18.3)  8 / 52 (15.4) | 1.23  1 | 0.50-3.03 | 0.653 |
| Thiopurines  Yes  No | 7 / 34 (20.6)  20 / 122 (16.4) | 1.32  1 | 0.51-3.45 | 0.567 |
| Use of biologics  Yes  No | 4 / 37 (10.8)  23 / 119 (19.3) | 0.51  1 | 0.16-1.57 | 0.232 |
| IBD-related surgical history  Yes  No | 1 / 6(16.7)  26 / 150 (17.3) | 0.95  1 | 0.11-8.51 | 0.966 |
| Hospitalization  Yes  No | 12 / 71 (16.9)  15 / 85 (17.6) | 0.95  1 | 0.41-2.19 | 0.902 |
| SRRS ≥ 150  Yes  No | 23 / 120 (19.2)  4 / 36 (11.1) | 1.90  1 | 0.61-5.90 | 0.262 |

**Supplementary Table 2.** Univariate analysis of factors associated with depression.

CD, Crohn’s disease; UC, ulcerative colitis, BMI, Body Mass Index; MAD, mood and/or anxiety disorders; IBD, inflammatory disease; EIM, extraintestinal manifestations; CRP, C-reactive protein;SRRS Social Readjustment Rating Scale; HADS, hospital anxiety and depression scale
