# Supplementary material for: Psychological burden and quality of life in newly diagnosed inflammatory bowel disease patients
Source: Front Psychol. 2024 Jan 29;15:1334308. doi: 10.3389/fpsyg.2024.1334308 (PMC10859525; doi:10.3389/fpsyg.2024.1334308)
Supplement: Supplementary file 3 [file Table_3.docx]

|  | **IBDQ32 < 160 / total**  n low QoL / n total (%) | **OR** | **95% confidence interval** | ***P*** |
| --- | --- | --- | --- | --- |
| Sex  Women  Men | 30 / 69 (43.5)  17 / 87 (19**.5**) | 3.17  1 | 1.55-6.46 | **0.001** |
| Age  <40 years  ≥40 years | 20 / 73 (27.4)  27 / 83 (32.5) | 1  1.28 | 0.64-2.55 | 0.486 |
| Disease Type  CD  UC | 34 / 80 (42.5)  13 / 76 (17.1) | 3.58  1 | 1.7-7.5 | **0.001** |
| BMI  <25  ≥25 | 33 / 88 (37.5)  14 / 68 (20.6) | 1  0.43 | 0.2-0.89 | **0.022** |
| Current smoker  Yes  No | 12 / 27 (44.4)  35 / 129 (27.1) | 2.15  1 | 0.92-5.04 | 0.075 |
| Marital status  Married /partner  Divorced/ Single/ Widowed | 29 / 94 (30.9)  18 / 62 (29) | 1.09  1 | 0.54-2.20 | 0.809 |
| Children  Yes  No | 32 / 92 (34.8)  15 / 64 (23.4) | 1.74  1 | 0.85-3.58 | 0.129 |
| Education  Low level  High level | 36 / 107 (33.6)  11 / 49 (22.4) | 1.75  1 | 0.80-3.83 | 0.157 |
| Active employment  Yes  No | 23 / 83 (27.7)  24 / 73 (32.9) | 1  1.28 | 0.64-2.53 | 0.483 |
| Comorbidity  Yes  No | 23 / 66 (34.8)  24 / 90 (26.7) | 1.47  1 | 0.74-2.93 | 0.271 |
| Previous history of MAD  Yes  No | 7 / 14 (50.0)  40 / 142 (28.2) | 2.55  1 | 0.84-7.73 | 0.089 |
| Active IBD  Mild  Moderate to Severe | 21 / 63 (33.3)  26 / 93 (28.0) | 1.29  1 | 0.64-2.57 | 0.473 |
| EIM  Yes  No | 6 / 19 (31.6)  41 / 137 (29.9) | 1.08  1 | 0.38-3.04 | 0.883 |
| Anemia  Yes  No | 11 / 47 (23.4)  36 / 109 (33.0) | 0.62  1 | 0.28-1.36 | 0.229 |
| CRP > 8 mg/L  Yes  No | 21 / 77 (27.3)  26 / 79 (32.9) | 0.76  1 | 0.38-1.52 | 0.443 |
| Fecal calprotectin > 250 µg/g  Yes  No | 30 / 98 (30.6)  17 / 58 (29.3) | 1.06  1 | 0.52-2.16 | 0.864 |
| Use of mesalazine  Yes  No | 25 / 110 (22.7)  22 / 46 (47.8) | 0.32  1 | 0.15-0.67 | **0.002** |
| Use of steroids  Yes  No | 38 / 104 (36.5)  9 / 52 (17.3) | 2.75  1 | 1.2-6.25 | **0.014** |
| Thiopurines  Yes  No | 11 /34 (32.4)  36 / 122 (29.5) | 1.14  1 | 0.50-2.59 | 0.749 |
| Use of biologics  Yes  No | 11 / 37 (29.7)  36 / 119 (30.3) | 0.97  1 | 0.44-2.18 | 0.952 |
| IBD-related surgical history  Yes  No | 1 / 6 (16.7)  46 / 150 (30.7) | 0.45  1 | 0.05-3.98 | 0.464 |
| Hospitalization  Yes  No | 23 / 71 (32.4)  24 / 85 (28.2) | 1.22  1 | 0.61-2.42 | 0.573 |
| SRRS ≥ 150  Yes  No | 43 / 120 (35.8)  4 / 36 (11.1) | 4.47  1 | 1.48-13.48 | **0.005** |

**Supplementary Table 3.** Univariate analysis of factors associated with quality of life.

CD, Crohn’s disease; UC, ulcerative colitis, BMI, Body Mass Index; MAD, mood and/or anxiety disorders; IBD, inflammatory disease; EIM, extraintestinal manifestations; CRP, C-reactive protein; SRRS Social Readjustment Rating Scale; IBDQ, Quality of life questionary for IBD
